# EM and SAGE Algorithms for DOA Estimation in the Presence of Unknown Uniform Noise

**DOI:** 10.3390/s23104811

**Published:** 2023-05-16

**Authors:** Ming-Yan Gong, Bin Lyu

**Affiliations:** 1 School of Information and Electronics, Beijing Institute of Technology, Beijing 100081, China; 2 Key Laboratory of Ministry of Education in Broadband Wireless Communication and Sensor Network Technology, Nanjing University of Posts and Telecommunications, Nanjing 210003, China; blyu@njupt.edu.cn

**Keywords:** array signal processing, DOA estimation, EM algorithm, maximum likelihood estimation, statistical signal processing

## Abstract

The existing expectation maximization (EM) and space-alternating generalized EM (SAGE) algorithms are only applied to direction of arrival (DOA) estimation in known noise. In this paper, the two algorithms are designed for DOA estimation in unknown uniform noise. Both the deterministic and random signal models are considered. In addition, a new modified EM (MEM) algorithm applicable to the noise assumption is also proposed. Next, these EM-type algorithms are improved to ensure the stability when the powers of sources are not equal. After being improved, simulation results illustrate that the EM algorithm has similar convergence with the MEM algorithm, the SAGE algorithm outperforms the EM and MEM algorithms for the deterministic signal model, and the SAGE algorithm cannot always outperform the EM and MEM algorithms for the random signal model. Furthermore, simulation results show that processing the same snapshots from the random signal model, the SAGE algorithm for the deterministic signal model can require the fewest computations.

## 1. Introduction

Direction of arrival (DOA) estimation is an important part of array signal processing and some high-resolution estimation techniques have been developed in the literature [1,2]. In particular, the maximum likelihood (ML) technique plays a critical role since it can offer the highest advantage in terms of both accuracy and spatial resolution. However, ML direction finding problems are non-convex and difficult to obtain their solutions in closed form.

One computationally efficient method to solve ML estimation problems is the classic expectation maximization (EM) algorithm [3,4], which has been employed for ML direction finding [5,6]. Each iteration of the EM algorithm is composed of an expectation step (E-step) and a maximization step (M-step). At the M-step, however, the EM algorithm updates all of the parameter estimates simultaneously, which causes slow convergence. In order to speed up the convergence of the EM algorithm, the space-alternating generalized EM (SAGE) algorithm has been proposed in [7]. References [8,9] show that the SAGE algorithm does yield faster convergence in terms of DOA estimation.

The existing EM and SAGE algorithms are usually derived under known noise [5,6,8,9]. The known noise is without unknown parameters, which may be unrealistic in certain applications. In fact, many seminal works in ML direction finding consider the so-called unknown uniform noise model [1,2,10,11,12], i.e., the noise covariance matrix can be expressed as $\tau I_{K}$, where τ is the only unknown noise parameter and $I_{K}$ is the K×K identity matrix. Under this noise assumption, a computationally attractive alternating projection algorithm is presented for computing the deterministic ML based DOA estimator in [10]. The authors in [11] investigate the statistical performance of this ML estimator and derive the Cramer–Rao lower bound. Moreover, some statistical properties of both the deterministic and random ML based DOA estimators under unknown uniform noise are compared in [12]. In addition to uniform noise, non-uniform noise has also attracted increasing attention [13,14,15,16]. The non-uniform noise has an arbitrary diagonal covariance matrix and, thus, makes DOA estimation complex. For efficiently computing both the deterministic and random ML based DOA estimators in unknown non-uniform noise, some feasible algorithms have been proposed in [17,18,19,20].

In this paper, we apply and design the EM and SAGE algorithms for DOA estimation in unknown uniform noise. Theoretical analyses indicate that for the deterministic signal model, τ has little effect on the two algorithms. However, the problem in the M-step of the EM algorithm for the random signal model can be no longer decomposed into parallel subproblems easily when τ is unknown. To proceed, we divide the M-step into two conditional M-steps (CM-steps) based on the expectation CM (ECM) algorithm [21]. In addition, we propose a new modified EM (MEM) algorithm applicable to the unknown uniform noise assumption. Note that although the EM algorithm in [22] is similar to the MEM algorithm, it is incorrectly derived. In brief, the proposed EM-type algorithms only need low-dimensional numerical searches at each iteration and are easy to perform. However, the proposed EM-type algorithms require accurate initial points, which is generally a computationally expensive task.

Existing simulations using EM-type algorithms always adopt sources of equal power [5,6,8,9,22]. We find, however, that when the powers of sources are unequal, the DOA estimates of multiple sources obtained by these EM-type algorithms tend to be consistent with the true DOA of the source with the largest power. To this end, we improve the proposed EM-type algorithms. After being improved, simulation results illustrate that (1) the EM algorithm has similar convergence with the MEM algorithm, (2) the SAGE algorithm outperforms the EM and MEM algorithms for the deterministic signal model, i.e., the SAGE algorithm converges faster and can avoid the convergence to an undesirable stationary point of the log-likelihood function (LLF) more efficiently, and (3) the SAGE algorithm cannot always outperform the EM and MEM algorithms for the random signal model.

The proposed EM-type algorithms for the deterministic signal model can process snapshots from the random signal model, so we, via simulation, compare these algorithms for both signal models. Simulations show that, under the same snapshots, initial DOA estimates, and stopping criterion, the SAGE algorithm for the deterministic signal model can require the fewest iterations and computations.

The contributions of this paper can be enumerated as follows:

- We apply and design the EM and SAGE algorithms for DOA estimation in unknown uniform noise. In particular, we derive the SAGE algorithm for random ML direction finding, which is not discussed in [7,8,22].
- We propose a new MEM algorithm applicable to the unknown uniform noise assumption.
- We improve these EM-type algorithms to ensure the stability when the powers of sources are not equal.
- Via simulation we show that the EM algorithm has similar convergence with the MEM algorithm and the SAGE algorithm outperforms the EM and MEM algorithms for the deterministic signal model. However, the SAGE algorithm cannot always outperform the EM and MEM algorithms for the random signal model.
- Via simulation we show that processing the same snapshots from the random signal model, the SAGE algorithm for the deterministic signal model can require the fewest iterations and computations.

The rest of this paper is outlined as follows: in Section 2, we formulate both the deterministic and random ML direction finding problems in unknown uniform noise. In Section 3, Section 4 and Section 5, we design the EM, MEM, and SAGE algorithms, respectively. We analyze some convergence properties of these EM-type algorithms in Section 6 and provide simulation results to compare the convergence of these EM-type algorithms in Section 7. Finally, we conclude this paper in Section 8.

## 2. Signal Model and Problem Statement

An array of *K* sensors is assumed to receive the plane waves emitted from *G* narrowband sources, which share the same known center wavelength χ. We use the Cartesian coordinate $\zeta_{k}={\left[x_{k}\;y_{k}\;z_{k}\right]}^{T}$ and the Spherical coordinate $\left(1,\mu_{g},\eta_{g}\right)$ to locate the *k*th sensor and the direction of the *g*th source, respectively. Here, ${\left[\cdot \right]}^{T}$ denotes transpose, $\mu_{g}$ and $\eta_{g}$ denote the elevation and azimuth angles of the *g*th source, respectively. For convenience, we transform $\left(1,\mu_{g},\eta_{g}\right)$ into the corresponding Cartesian coordinate $\gamma_{g}={\left[\sin\left(\mu_{g}\right)\cos\left(\eta_{g}\right)\;\sin\left(\mu_{g}\right)\sin\left(\eta_{g}\right)\;\cos\left(\mu_{g}\right)\right]}^{T}$. Let the origin be the reference point such that the signal received at the array is written as

(1) $$\begin{array}{c} w\left(t\right)=\sum_{g=1}^{G}b\left(\xi_{g}\right)m_{g}\left(t\right)+v\left(t\right)=B\left(\xi \right)m\left(t\right)+v\left(t\right), \end{array}$$

where $b\left(\xi_{g}\right)={\left[e^{𝚥\phi_{1,g}}\;\cdots \;e^{𝚥\phi_{K,g}}\right]}^{T}$ with DOA $\xi_{g}=\left(\mu_{g},\eta_{g}\right)\in \Omega$, $\phi_{k,g}=-\frac{2\pi }{\chi }\zeta_{k}^{T}\gamma_{g}$, and $𝚥=\sqrt{-1}$, $m_{g}\left(t\right)$ is the signal of the *g*th source, and v(t) is complex Gaussian noise of zero mean and covariance $\tau I_{K}$, i.e., $v\left(t\right)∼\mathrm{CN}\left(0,\tau I_{K}\right)$ with $0={\left[0\;\cdots \;0\right]}^{T}$. In (1), $B\left(\xi \right)=[b\left(\xi_{1}\right)\;\cdots \;b\left(\xi_{G}\right)]$, $\xi =(\xi_{1},\cdots ,\xi_{G})\in \Omega$ with $\Omega =\Omega^{G}$, and $m\left(t\right)={\left[m_{1}\left(t\right)\;\cdots \;m_{G}\left(t\right)\right]}^{T}$.

EM-type algorithms need to define the unavailable complete data. According to the classic EM paradigm for superimposed signals [5,6], we construct *L* independent snapshots, the incomplete data of the EM algorithm, by 

(2) $$\begin{array}{c} w\left(t\right)=\sum_{g=1}^{G}\left[b\left(\xi_{g}\right)m_{g}\left(t\right)+v_{g}\left(t\right)\right]=\sum_{g=1}^{G}h_{g}\left(t\right),t=1,2,\cdots ,L, \end{array}$$

where the $h_{g}\left(t\right)$’s are the complete data. Moreover, the $v_{g}\left(t\right)$’s are mutually uncorrelated and $v_{g}\left(t\right)∼\mathrm{CN}\left(0,\beta_{g}\tau I_{K}\right)$, where $\beta ={\left[\beta_{1}\;\cdots \;\beta_{G}\right]}^{T}>0$ and $1^{T}\beta =1$ with $1={\left[1\;\cdots \;1\right]}^{T}$. Note that the incomplete- and complete-data LLFs require the distributions of the $m_{g}\left(t\right)$’s, we adopt the following two statistical models separately.

### 2.1. Deterministic Signal Model

We let the $m_{g}\left(t\right)$’s be deterministic and unknown [5,6,10,11], which leads to $h_{g}\left(t\right)∼\mathrm{CN}\left(b\left(\xi_{g}\right)m_{g}\left(t\right),\beta_{g}\tau I_{K}\right)$ and $w\left(t\right)∼\mathrm{CN}\left(B\left(\xi \right)m\left(t\right),\tau I_{K}\right)$. Then, the incomplete- and complete-data LLFs are formulated by

(3) $$\begin{array}{ccc} J(\Psi ,\tau ) & = & \sum_{t=1}^{L}\log p\left(w(t);\xi ,m(t),\tau \right)\\ & = & -LK\log\left(\pi \tau \right)-\frac{1}{\tau }\sum_{t=1}^{L}{∥w\left(t\right)-B\left(\xi \right)m\left(t\right)∥}^{2}, \end{array}$$

(4) $$\begin{array}{ccc} Z(\Psi ,\tau ) & = & \sum_{t=1}^{L}\sum_{g=1}^{G}\log p\left(h_{g}\left(t\right);\xi_{g},m_{g}\left(t\right),\tau \right)\\ & = & -LKG\log\left(\pi \tau \right)-LK\sum_{g=1}^{G}\log\left(\beta_{g}\right)-\frac{1}{\tau }\sum_{t=1}^{L}\sum_{g=1}^{G}\frac{1}{\beta_{g}}{∥h_{g}\left(t\right)-b\left(\xi_{g}\right)m_{g}\left(t\right)∥}^{2}, \end{array}$$

where ∥·∥ denotes Euclidean norm and M=[m(1)⋯m(L)]. Note that Ψ=(ξ,M) denotes the signal parameters while τ is the only noise parameter. Finally, the ML estimation problem is

(5) $$\begin{array}{c} \max_{\xi \in \Omega ,M,\tau >0}J\left(\Psi ,\tau \right). \end{array}$$

### 2.2. Random Signal Model

We assume $m_{g}\left(t\right)∼\mathrm{CN}\left(0,\rho_{g}\right)$ where $\rho_{g}$ is the power of the *g*th source. For simplicity, all of the $m_{g}\left(t\right)$’s and $v_{g}\left(t\right)$’s are assumed to be mutually uncorrelated [5,6]. Next, we have $h_{g}\left(t\right)∼\mathrm{CN}\left(0,N_{g}\right)$ with $N_{g}=\rho_{g}b\left(\xi_{g}\right)b^{H}\left(\xi_{g}\right)+\beta_{g}\tau I_{K}$, where ${\left[\cdot \right]}^{H}$ denotes conjugate transpose, and $w\left(t\right)∼\mathrm{CN}\left(0,N_{w}\right)$ with $N_{w}=\sum_{g=1}^{G}N_{g}$. The incomplete- and complete-data LLFs are formulated by

(6) $$\begin{array}{ccc} J(\Psi ,\tau ) & = & \sum_{t=1}^{L}\log p\left(w(t);\xi ,\rho ,\tau \right)\\ & = & -L\left[K\log\left(\pi \right)+\log\left(\mathrm{Det}(N_{w})\right)+\mathrm{Tr}\left(N_{w}^{-1}{\hat{P}}_{w}\right)\right], \end{array}$$

(7) $$\begin{array}{ccc} Z(\Psi ,\tau ) & = & \sum_{t=1}^{L}\sum_{g=1}^{G}\log p\left(h_{g}\left(t\right);\xi_{g},\rho_{g},\tau \right)\\ & = & -L\sum_{g=1}^{G}\left[K\log\left(\pi \right)+\log\left(\mathrm{Det}(N_{g})\right)+\mathrm{Tr}\left(N_{g}^{-1}{\hat{P}}_{g}\right)\right], \end{array}$$

where ${\left[\cdot \right]}^{-1}$, Det(·), and Tr(·) denote inversion, determinant, and trace, respectively. Moreover, Ψ=(ξ,ρ), ${\hat{P}}_{w}=\left(1/L\right)\sum_{t=1}^{L}w\left(t\right)w^{H}\left(t\right)$, ${\hat{P}}_{g}=\left(1/L\right)\sum_{t=1}^{L}h_{g}\left(t\right)h_{g}^{H}\left(t\right)$, and $\rho ={\left[\rho_{1}\;\cdots \;\rho_{G}\right]}^{T}$. Finally, the ML estimation problem is

(8) $$\begin{array}{c} \max_{\xi \in \Omega ,\rho \geq 0,\tau >0}J\left(\Psi ,\tau \right). \end{array}$$

## 3. EM Algorithm

In this section, we design and derive the EM algorithm for solving problems (5) and (8). The E- and M-steps at the *r*th iteration are introduced below. Let ${\left[\cdot \right]}^{\left(r\right)}$, E{·}, and D{·} denote an iterative value at the *r*th iteration, expectation, and covariance, respectively. ${\left[\cdot \right]}^{\left(0\right)}$ is an initial estimate.

### 3.1. Deterministic Signal Model

#### 3.1.1. E-Step

Calculate the conditional expectation of the complete-data LLF in (4)

(9) $$\begin{array}{ccc} E\left\{Z\left(\Delta \right)|W;\Delta^{\left(r-1\right)}\right\} & = & C-L\{KG\log\left(\tau \right)+K\sum_{g=1}^{G}\log\left(\beta_{g}\right)+\frac{1}{\tau }[u^{\left(r\right)}+\\ & \; & \frac{1}{L}\sum_{t=1}^{L}\sum_{g=1}^{G}\frac{1}{\beta_{g}}{∥h_{g}^{\left(r\right)}\left(t\right)-b\left(\xi_{g}\right)m_{g}\left(t\right)∥}^{2}]\}, \end{array}$$

where Δ=(Ψ,τ)=(ξ,M,τ), W=[w(1)⋯w(L)], and C=−LKGlog(π). In (9), the conditional distribution of $h_{g}\left(t\right)$ can be derived from [23] and

(10) $$\begin{array}{ccc} h_{g}^{\left(r\right)}\left(t\right) & = & E\left\{h_{g}\left(t\right)|W;\Delta^{\left(r-1\right)}\right\}\\ & = & b\left(\xi_{g}^{\left(r-1\right)}\right)m_{g}^{\left(r-1\right)}\left(t\right)+\beta_{g}\left[w\left(t\right)-B\left(\xi^{\left(r-1\right)}\right)m^{\left(r-1\right)}\left(t\right)\right], \end{array}$$

(11) $$\begin{array}{ccc} u^{\left(r\right)} & = & \frac{1}{L}\sum_{t=1}^{L}\sum_{g=1}^{G}\frac{1}{\beta_{g}}\mathrm{Tr}\left(D\left\{h_{g}\left(t\right)|W;\Delta^{\left(r-1\right)}\right\}\right)=\frac{1}{L}\sum_{t=1}^{L}\sum_{g=1}^{G}\frac{1}{\beta_{g}}\mathrm{Tr}\left(\beta_{g}\left(1-\beta_{g}\right)\tau^{\left(r-1\right)}I_{K}\right)\\ & = & K\left(G-1\right)\tau^{\left(r-1\right)}. \end{array}$$

#### 3.1.2. M-Step

Update the estimates of Ψ and τ by solving

(12) $$\begin{array}{c} \min_{\xi \in \Omega ,M,\tau >0}KG\log\left(\tau \right)+\frac{1}{\tau }\left[u^{\left(r\right)}+\frac{1}{L}\sum_{t=1}^{L}\sum_{g=1}^{G}\frac{1}{\beta_{g}}∥h_{g}^{\left(r\right)}\left(t\right)-b\left(\xi_{g}\right)m_{g}\left(t\right){∥}^{2}\right]. \end{array}$$

$\Psi^{\left(r\right)}=\left(\xi^{\left(r\right)},M^{\left(r\right)}\right)$ and $\tau^{\left(r\right)}$ are obtained by [6]

(13) $$\begin{array}{ccc} \xi_{g}^{\left(r\right)} & = & \arg\max_{\xi_{g}\in \Omega }\mathrm{Tr}\left(\Gamma_{g}{\hat{P}}_{g}^{\left(r\right)}\right),\forall g, \end{array}$$

(14) $$\begin{array}{ccc} m_{g}^{\left(r\right)}\left(t\right) & = & b^{H}\left(\xi_{g}^{\left(r\right)}\right)h_{g}^{\left(r\right)}\left(t\right)/K,\forall g,t, \end{array}$$

(15) $$\begin{array}{ccc} \tau^{\left(r\right)} & = & \left(1-1/G\right)\tau^{\left(r-1\right)}+\left(1/K/G\right)\sum_{g=1}^{G}d_{g}^{\left(r\right)}/\beta_{g}, \end{array}$$

where $\Gamma_{g}=b\left(\xi_{g}\right)b^{H}\left(\xi_{g}\right)/K$, ${\hat{P}}_{g}^{\left(r\right)}=\left(1/L\right)\sum_{t=1}^{L}h_{g}^{\left(r\right)}\left(t\right){\left[h_{g}^{\left(r\right)}\left(t\right)\right]}^{H}$, and $d_{g}^{\left(r\right)}=\mathrm{Tr}\left(\left(I_{K}-\Gamma_{g}^{\left(r\right)}\right){\hat{P}}_{g}^{\left(r\right)}\right)\geq 0$. In (15), $\tau^{\left(r\right)}>0$ if $\tau^{\left(r-1\right)}>0$.

**Remark** **1.** *Note that the $h_{g}^{\left(r\right)}\left(t\right)$’s in (10), the $\xi_{g}^{\left(r\right)}$’s in (13), and the $m_{g}^{\left(r\right)}\left(t\right)$’s in (14) are unrelated to $\tau^{\left(r-1\right)}$, we can omit (15) due to the nuisance parameter τ.*

### 3.2. Random Signal Model

#### 3.2.1. E-Step

Calculate the conditional expectation of the complete-data LLF in (7)

(16) $$\begin{array}{c} E\left\{Z\left(\Delta \right)|W;\Delta^{\left(r-1\right)}\right\}=C-L\sum_{g=1}^{G}\left[\log\left(\mathrm{Det}(N_{g})\right)+\mathrm{Tr}\left(N_{g}^{-1}{\hat{P}}_{g}^{\left(r\right)}\right)\right], \end{array}$$

where Δ=(Ψ,τ)=(ξ,ρ,τ) and

(17) $$\begin{array}{ccc} {\hat{P}}_{g}^{\left(r\right)} & = & E\left\{{\hat{P}}_{g}|W;\Delta^{\left(r-1\right)}\right\}\\ & = & N_{g}^{\left(r-1\right)}-N_{g}^{\left(r-1\right)}{\left[N_{w}^{\left(r-1\right)}\right]}^{-1}N_{g}^{\left(r-1\right)}+N_{g}^{\left(r-1\right)}{\left[N_{w}^{\left(r-1\right)}\right]}^{-1}{\hat{P}}_{w}{\left[N_{w}^{\left(r-1\right)}\right]}^{-1}N_{g}^{\left(r-1\right)}. \end{array}$$

#### 3.2.2. M-Step

Update the estimates of Ψ and τ by solving

(18) $$\begin{array}{c} \min_{\xi \in \Omega ,\rho \geq 0,\tau >0}\sum_{g=1}^{G}\left[\log\left(\mathrm{Det}(N_{g})\right)+\mathrm{Tr}\left(N_{g}^{-1}{\hat{P}}_{g}^{\left(r\right)}\right)\right], \end{array}$$

which is difficult to be decomposed into parallel subproblems due to τ. To obtain $\Psi^{\left(r\right)}$ and $\tau^{\left(r\right)}$ easily, we rewrite (18) as

(19) $$\begin{array}{c} \min_{\xi \in \Omega ,\sigma \geq 0,\tau >0}KG\log\left(\tau \right)+\sum_{g=1}^{G}\left[\log\left(\mathrm{Det}(Q_{g})\right)+\frac{1}{\tau }\mathrm{Tr}\left(Q_{g}^{-1}{\hat{P}}_{g}^{\left(r\right)}\right)\right], \end{array}$$

where $N_{g}=\tau Q_{g}$ with $Q_{g}=\sigma_{g}b\left(\xi_{g}\right)b^{H}\left(\xi_{g}\right)+\beta_{g}I_{K}$ and $\sigma ={\left[\sigma_{1}\;\cdots \;\sigma_{G}\right]}^{T}$ with $\sigma_{g}=\rho_{g}/\tau$. We now divide the M-step into the following two CM-steps based on the ECM algorithm [21], i.e., the algorithm becomes the ECM algorithm. For convenience, we still call it the EM algorithm.

- *First CM-step:* Estimate Ψ but hold $\tau =\tau^{\left(r-1\right)}$ fixed. Then, problem (19) can be decomposed into the *G* parallel subproblems
  (20) $$\begin{array}{c} \min_{\xi_{g}\in \Omega ,\sigma_{g}\geq 0}\log\left(\mathrm{Det}(Q_{g})\right)+\frac{1}{\tau^{\left(r-1\right)}}\mathrm{Tr}\left(Q_{g}^{-1}{\hat{P}}_{g}^{\left(r\right)}\right),\forall g. \end{array}$$
  $\Psi^{\left(r\right)}=\left(\xi^{\left(r\right)},\rho^{\left(r\right)}\right)$ is obtained by [6]
  (21) $$\begin{array}{ccc} \xi_{g}^{\left(r\right)} & = & \arg\max_{\xi_{g}\in \Omega }\mathrm{Tr}\left(\Gamma_{g}{\hat{P}}_{g}^{\left(r\right)}\right),\forall g, \end{array}$$
  (22) $$\begin{array}{ccc} \rho_{g}^{\left(r\right)} & = & \sigma_{g}^{\left(r\right)}\tau^{\left(r-1\right)}=\max\left\{\left(e_{g}^{\left(r\right)}-\beta_{g}\tau^{\left(r-1\right)}\right)/K,0\right\},\forall g, \end{array}$$
  where $e_{g}^{\left(r\right)}=\mathrm{Tr}\left(\Gamma_{g}^{\left(r\right)}{\hat{P}}_{g}^{\left(r\right)}\right)$ and $\xi_{g}^{\left(r\right)}$ is indeterminate if $\rho_{g}^{\left(r\right)}=0$.
- *Second CM-step:* Estimate τ but hold $\Psi =\Psi^{\left(r\right)}$ fixed. Then, problem (19) is simplified to
  (23) $$\begin{array}{c} \min_{\tau >0}KG\log\left(\tau \right)+\frac{1}{\tau }\sum_{g=1}^{G}\mathrm{Tr}\left({\left[Q_{g}^{\left(r\right)}\right]}^{-1}{\hat{P}}_{g}^{\left(r\right)}\right). \end{array}$$
  $\tau^{\left(r\right)}$ is obtained by
  (24) $$\begin{array}{c} \tau^{\left(r\right)}=\frac{1}{KG}\sum_{g=1}^{G}\mathrm{Tr}\left({\left[Q_{g}^{\left(r\right)}\right]}^{-1}{\hat{P}}_{g}^{\left(r\right)}\right)=\frac{1}{K}\tau^{\left(r-1\right)}+\frac{1}{KG}\sum_{g=1}^{G}d_{g}^{\left(r\right)}/\beta_{g}, \end{array}$$
  where $d_{g}^{\left(r\right)}=\mathrm{Tr}\left(\left(I_{K}-\Gamma_{g}^{\left(r\right)}\right){\hat{P}}_{g}^{\left(r\right)}\right)\geq 0$ and $\tau^{\left(r\right)}>0$ if $\tau^{\left(r-1\right)}>0$.

## 4. MEM Algorithm

In the previous section, β is fixed and known. In this section, we regard β as a parameter to be estimated and, thus, propose an MEM algorithm applicable to the unknown uniform noise assumption.

To estimate τ and β easily, we introduce $\tau_{g}=\beta_{g}\tau$ as the common noise variance of the *g*th source and have

(25) $$\begin{array}{c} v_{g}\left(t\right)∼\mathrm{CN}\left(0,\tau_{g}I_{K}\right). \end{array}$$

Clearly, $\tau =\sum_{g=1}^{G}\tau_{g}$ and $\beta_{g}=\tau_{g}/\tau$. The E- and M-steps at the *r*th iteration are introduced below. Let $\tau ={\left[\tau_{1}\;\cdots \;\tau_{G}\right]}^{T}$.

### 4.1. Deterministic Signal Model

Based on (25), the complete-data LLF in (4) is rewritten as

(26) $$\begin{array}{c} Z\left(\Delta \right)=C-LK\sum_{g=1}^{G}\log\left(\tau_{g}\right)-\sum_{t=1}^{L}\sum_{g=1}^{G}\frac{1}{\tau_{g}}{∥h_{g}\left(t\right)-b\left(\xi_{g}\right)m_{g}\left(t\right)∥}^{2}, \end{array}$$

where Δ=(Ψ,τ)=(ξ,M,τ) and $h_{g}\left(t\right)∼\mathrm{CN}\left(b\left(\xi_{g}\right)m_{g}\left(t\right),\tau_{g}I_{K}\right)$.

#### 4.1.1. E-Step

Calculate the conditional expectation of the complete-data LLF in (26)

(27) $$\begin{array}{ccc} E\left\{Z\left(\Delta \right)|W;\Delta^{\left(r-1\right)}\right\} & = & C-L\sum_{g=1}^{G}\{K\log\left(\tau_{g}\right)+\frac{1}{\tau_{g}}[u_{g}^{\left(r\right)}+\\ & \; & \frac{1}{L}\sum_{t=1}^{L}{∥h_{g}^{\left(r\right)}\left(t\right)-b\left(\xi_{g}\right)m_{g}\left(t\right)∥}^{2}]\}, \end{array}$$

where

(28) $$\begin{array}{ccc} h_{g}^{\left(r\right)}\left(t\right) & = & E\left\{h_{g}\left(t\right)|W;\Delta^{\left(r-1\right)}\right\}\\ & = & b\left(\xi_{g}^{\left(r-1\right)}\right)m_{g}^{\left(r-1\right)}\left(t\right)+\left(\tau_{g}^{\left(r-1\right)}/\tau^{\left(r-1\right)}\right)\left[w\left(t\right)-B\left(\xi^{\left(r-1\right)}\right)m^{\left(r-1\right)}\left(t\right)\right], \end{array}$$

(29) $$\begin{array}{ccc} u_{g}^{\left(r\right)} & = & \frac{1}{L}\sum_{t=1}^{L}\mathrm{Tr}\left(D\left\{h_{g}\left(t\right)|W;\Delta^{\left(r-1\right)}\right\}\right)=K\tau_{g}^{\left(r-1\right)}\left(1-\tau_{g}^{\left(r-1\right)}/\tau^{\left(r-1\right)}\right). \end{array}$$

#### 4.1.2. M-Step

Update the estimates of Ψ and τ by solving the *G* parallel subproblems

(30) $$\begin{array}{c} \min_{\xi_{g}\in \Omega ,m_{g},\tau_{g}>0}K\log\left(\tau_{g}\right)+\frac{1}{\tau_{g}}\left[u_{g}^{\left(r\right)}+\frac{1}{L}\sum_{t=1}^{L}∥h_{g}^{\left(r\right)}\left(t\right)-b\left(\xi_{g}\right)m_{g}\left(t\right){∥}^{2}\right],\forall g, \end{array}$$

where $m_{g}=\left[m_{g}\left(1\right)\;\cdots \;m_{g}\left(L\right)\right]$. $\Psi^{\left(r\right)}=\left(\xi^{\left(r\right)},M^{\left(r\right)}\right)$ and $\tau^{\left(r\right)}$ are obtained by

(31) $$\begin{array}{ccc} \xi_{g}^{\left(r\right)} & = & \arg\max_{\xi_{g}\in \Omega }\mathrm{Tr}\left(\Gamma_{g}{\hat{P}}_{g}^{\left(r\right)}\right),\forall g, \end{array}$$

(32) $$\begin{array}{ccc} m_{g}^{\left(r\right)}\left(t\right) & = & b^{H}\left(\xi_{g}^{\left(r\right)}\right)h_{g}^{\left(r\right)}\left(t\right)/K,\forall g,t, \end{array}$$

(33) $$\begin{array}{ccc} \tau_{g}^{\left(r\right)} & = & \tau_{g}^{\left(r-1\right)}\left(1-\tau_{g}^{\left(r-1\right)}/\tau^{\left(r-1\right)}\right)+d_{g}^{\left(r\right)}/K,\forall g, \end{array}$$

where ${\hat{P}}_{g}^{\left(r\right)}=\left(1/L\right)\sum_{t=1}^{L}h_{g}^{\left(r\right)}\left(t\right){\left[h_{g}^{\left(r\right)}\left(t\right)\right]}^{H}$ and $d_{g}^{\left(r\right)}=\mathrm{Tr}\left(\left(I_{K}-\Gamma_{g}^{\left(r\right)}\right){\hat{P}}_{g}^{\left(r\right)}\right)\geq 0$. In (33), if $\tau^{\left(r-1\right)}>0$, we have $1-\tau_{g}^{\left(r-1\right)}/\tau^{\left(r-1\right)}>0,\forall g$, and then $\tau_{g}^{\left(r\right)}>0,\forall g$, i.e., $\tau^{\left(r\right)}>0$.

### 4.2. Random Signal Model

Based on (25), the complete-data LLF in (7) is rewritten as

(34) $$\begin{array}{c} Z\left(\Delta \right)=C-L\sum_{g=1}^{G}\left[\log\left(\mathrm{Det}(N_{g})\right)+\mathrm{Tr}\left(N_{g}^{-1}{\hat{P}}_{g}\right)\right], \end{array}$$

where Δ=(Ψ,τ)=(ξ,ρ,τ) and $N_{g}=\rho_{g}b\left(\xi_{g}\right)b^{H}\left(\xi_{g}\right)+\tau_{g}I_{K}$.

#### 4.2.1. E-Step

Calculate the conditional expectation of the complete-data LLF in (34)

(35) $$\begin{array}{c} E\left\{Z\left(\Delta \right)|W;\Delta^{\left(r-1\right)}\right\}=C-L\sum_{g=1}^{G}\left[\log\left(\mathrm{Det}(N_{g})\right)+\mathrm{Tr}\left(N_{g}^{-1}{\hat{P}}_{g}^{\left(r\right)}\right)\right], \end{array}$$

where

(36) $$\begin{array}{ccc} {\hat{P}}_{g}^{\left(r\right)} & = & E\left\{{\hat{P}}_{g}|W;\Delta^{\left(r-1\right)}\right\}\\ & = & N_{g}^{\left(r-1\right)}-N_{g}^{\left(r-1\right)}{\left[N_{w}^{\left(r-1\right)}\right]}^{-1}N_{g}^{\left(r-1\right)}+N_{g}^{\left(r-1\right)}{\left[N_{w}^{\left(r-1\right)}\right]}^{-1}{\hat{P}}_{w}{\left[N_{w}^{\left(r-1\right)}\right]}^{-1}N_{g}^{\left(r-1\right)}. \end{array}$$

#### 4.2.2. M-Step

Update the estimates of Ψ and τ by solving the *G* parallel subproblems

(37) $$\begin{array}{c} \min_{\xi_{g}\in \Omega ,\sigma_{g}\geq 0,\tau_{g}>0}K\log\left(\tau_{g}\right)+\log\left(\mathrm{Det}(Q_{g})\right)+\frac{1}{\tau_{g}}\mathrm{Tr}\left(Q_{g}^{-1}{\hat{P}}_{g}^{\left(r\right)}\right),\forall g, \end{array}$$

where $N_{g}=\tau_{g}Q_{g}$ with $Q_{g}=\sigma_{g}b\left(\xi_{g}\right)b^{H}\left(\xi_{g}\right)+I_{K}$ and $\sigma_{g}=\rho_{g}/\tau_{g}$. Since $\mathrm{Det}\left(Q_{g}\right)=K\sigma_{g}+1$ and $Q_{g}^{-1}=I_{K}-\frac{K\sigma_{g}}{K\sigma_{g}+1}\Gamma_{g}$, subproblems (37) are rewritten as

(38) $$\begin{array}{c} \min_{\xi_{g}\in \Omega ,\sigma_{g}\geq 0,\tau_{g}>0}K\log\left(\tau_{g}\right)+\log\left(K\sigma_{g}+1\right)+\frac{1}{\tau_{g}}\mathrm{Tr}\left({\hat{P}}_{g}^{\left(r\right)}\right)-\frac{K\sigma_{g}}{\tau_{g}\left(K\sigma_{g}+1\right)}\mathrm{Tr}\left(\Gamma_{g}{\hat{P}}_{g}^{\left(r\right)}\right),\forall g. \end{array}$$

To proceed, we first eliminate $\sigma ={\left[\sigma_{1}\;\cdots \;\sigma_{G}\right]}^{T}$ in (38) [24,25]. Thus, when obtaining $\xi^{\left(r\right)}$ and $\tau^{\left(r\right)}$, $\sigma^{\left(r\right)}$ and $\rho^{\left(r\right)}$ are obtained by [6]

(39) $$\begin{array}{ccc} \sigma_{g}^{\left(r\right)} & = & \max\left\{\left(e_{g}^{\left(r\right)}/\tau_{g}^{\left(r\right)}-1\right)/K,0\right\},\forall g, \end{array}$$

(40) $$\begin{array}{ccc} \rho_{g}^{\left(r\right)} & = & \sigma_{g}^{\left(r\right)}\tau_{g}^{\left(r\right)}=\max\left\{\left(e_{g}^{\left(r\right)}-\tau_{g}^{\left(r\right)}\right)/K,0\right\},\forall g, \end{array}$$

where $e_{g}^{\left(r\right)}=\mathrm{Tr}\left(\Gamma_{g}^{\left(r\right)}{\hat{P}}_{g}^{\left(r\right)}\right)$. Note that if $\sigma_{g}^{\left(r\right)}=0$, $\xi_{g}^{\left(r\right)}$ is indeterminate and $\tau_{g}^{\left(r\right)}=\mathrm{Tr}\left({\hat{P}}_{g}^{\left(r\right)}\right)/K$ by (38). To obtain $\xi^{\left(r\right)}$ and $\tau^{\left(r\right)}$, we assume $\sigma^{\left(r\right)}>0$. After eliminating σ, subproblems (38) are simplified to

(41) $$\begin{array}{c} \min_{\xi_{g}\in \Omega ,\tau_{g}>0}\left(K-1\right)\log\left(\tau_{g}\right)+\log\left(\mathrm{Tr}(\Gamma_{g}{\hat{P}}_{g}^{\left(r\right)})\right)+\frac{1}{\tau_{g}}\mathrm{Tr}\left(\left(I_{K}-\Gamma_{g}\right){\hat{P}}_{g}^{\left(r\right)}\right),\forall g. \end{array}$$

Next, we eliminate τ in (41). Thus, when obtaining $\xi^{\left(r\right)}$, $\tau^{\left(r\right)}$ is obtained by $\tau_{g}^{\left(r\right)}=d_{g}^{\left(r\right)}/\left(K-1\right),\forall g$, where $d_{g}^{\left(r\right)}=\mathrm{Tr}\left(\left(I_{K}-\Gamma_{g}^{\left(r\right)}\right){\hat{P}}_{g}^{\left(r\right)}\right)$. After eliminating τ, subproblems (41) are simplified to

(42) $$\begin{array}{c} \min_{\xi_{g}\in \Omega }\left(K-1\right)\log\left(\mathrm{Tr}\left({\hat{P}}_{g}^{\left(r\right)}\right)-\mathrm{Tr}\left(\Gamma_{g}{\hat{P}}_{g}^{\left(r\right)}\right)\right)+\log\left(\mathrm{Tr}(\Gamma_{g}{\hat{P}}_{g}^{\left(r\right)})\right),\forall g, \end{array}$$

where $\xi_{g}\in \delta_{g}^{\left(r\right)}=\left\{\xi_{g}\in \Omega |\mathrm{Tr}\left(\Gamma_{g}{\hat{P}}_{g}^{\left(r\right)}\right)>\mathrm{Tr}\left({\hat{P}}_{g}^{\left(r\right)}\right)/K\right\}$ due to the fact that when $\xi_{g}^{\left(r\right)}\in \delta_{g}^{\left(r\right)}$, $e_{g}^{\left(r\right)}=\mathrm{Tr}\left(\Gamma_{g}^{\left(r\right)}{\hat{P}}_{g}^{\left(r\right)}\right)>\mathrm{Tr}\left({\hat{P}}_{g}^{\left(r\right)}\right)/K$ and

(43) $$\begin{array}{cc} & \sigma_{g}^{\left(r\right)}=\max\left\{\left(e_{g}^{\left(r\right)}/\tau_{g}^{\left(r\right)}-1\right)/K,0\right\}=\left(e_{g}^{\left(r\right)}-\mathrm{Tr}\left({\hat{P}}_{g}^{\left(r\right)}\right)/K\right)/d_{g}^{\left(r\right)}>0,\forall g. \end{array}$$

Since $\left(K-1\right)\log\left(\mathrm{Tr}({\hat{P}}_{g}^{\left(r\right)})-x\right)+\log\left(x\right)$ is a monotonically decreasing function of *x* for $x\geq \mathrm{Tr}({\hat{P}}_{g}^{\left(r\right)})/K$, subproblems (42) are equivalent to

(44) $$\begin{array}{c} \max_{\xi_{g}\in \delta_{g}^{\left(r\right)}}\mathrm{Tr}\left(\Gamma_{g}{\hat{P}}_{g}^{\left(r\right)}\right),\forall g. \end{array}$$

Based on the above analysis, $\Psi^{\left(r\right)}=\left(\xi^{\left(r\right)},\rho^{\left(r\right)}\right)$ and $\tau^{\left(r\right)}$ are obtained by

(45) $$\begin{array}{ccc} \xi_{g}^{\left(r\right)} & = & \arg\max_{\xi_{g}\in \Omega }\mathrm{Tr}\left(\Gamma_{g}{\hat{P}}_{g}^{\left(r\right)}\right),\forall g, \end{array}$$

(46) $$\begin{array}{ccc} \tau_{g}^{\left(r\right)} & = & \left\{\begin{array}{cc} d_{g}^{\left(r\right)}/\left(K-1\right)\geq 0, & e_{g}^{\left(r\right)}>\mathrm{Tr}\left({\hat{P}}_{g}^{\left(r\right)}\right)/K,\\ \mathrm{Tr}({\hat{P}}_{g}^{\left(r\right)})/K\geq 0, & e_{g}^{\left(r\right)}\leq \mathrm{Tr}\left({\hat{P}}_{g}^{\left(r\right)}\right)/K, \end{array}\right.\forall g, \end{array}$$

(47) $$\begin{array}{ccc} \rho_{g}^{\left(r\right)} & = & \max\left\{\left(e_{g}^{\left(r\right)}-\mathrm{Tr}\left({\hat{P}}_{g}^{\left(r\right)}\right)/K\right)/\left(K-1\right),0\right\},\forall g, \end{array}$$

where we can use a proof by contradiction to verify that $\tau^{\left(r\right)}>0$ if $\tau^{\left(r-1\right)}>0$.

## 5. SAGE Algorithm

In the SAGE algorithm, each iteration consists of *G* cycles and $\xi_{q}^{\left(r\right)}$ is obtained at the *q*th cycle of the *r*th iteration. Let ${\left[\cdot \right]}^{\left(r,q\right)}$ mean an iterative value at the *q*th cycle of the *r*th iteration, ${\left[\cdot \right]}^{\left(r-1\right)}={\left[\cdot \right]}^{\left(r-1,G\right)}={\left[\cdot \right]}^{\left(r,0\right)}$.

At the *q*th cycle of the *r*th iteration, the SAGE algorithm first constructs the complete data by [7,8]

(48) $$h_{g}\left(t\right)=\left\{\begin{array}{cc} b\left(\xi_{g}\right)m_{g}\left(t\right)+v\left(t\right) & g=q,\\ b\left(\xi_{g}\right)m_{g}\left(t\right) & g\neq q. \end{array}\right.$$

Then, the E- and M-steps at the *q*th cycle of the *r*th iteration are introduced below.

### 5.1. Deterministic Signal Model

Based on (48), we have $h_{q}\left(t\right)∼\mathrm{CN}\left(b\left(\xi_{q}\right)m_{q}\left(t\right),\tau I_{K}\right)$ and the $h_{g}\left(t\right)$’s with g≠q are deterministic. The complete-data LLF is expressed as

(49) $$\begin{array}{ccc} Z(\xi_{q},m_{q},\tau ) & = & \sum_{t=1}^{L}\log p\left(h_{q}\left(t\right);\xi_{q},m_{q}\left(t\right),\tau \right)\\ & = & -LK\log\left(\pi \tau \right)-\frac{1}{\tau }\sum_{t=1}^{L}{∥h_{q}\left(t\right)-b\left(\xi_{q}\right)m_{q}\left(t\right)∥}^{2}. \end{array}$$

#### 5.1.1. E-Step

Calculate the conditional expectation of the complete-data LLF in (49)

(50) $$\begin{array}{c} E\left\{Z\left(\xi_{q},m_{q},\tau \right)|W;\Delta^{\left(r,q-1\right)}\right\}=-LK\log\left(\pi \tau \right)-\frac{1}{\tau }\sum_{t=1}^{L}{∥h_{q}^{\left(r\right)}\left(t\right)-b\left(\xi_{q}\right)m_{q}\left(t\right)∥}^{2}, \end{array}$$

where Δ=(ξ,M,τ), $0_{K}$ is the K×K zero matrix, and 

(51) $$\begin{array}{ccc} h_{q}^{\left(r\right)}\left(t\right) & = & h_{q}^{\left(r,q\right)}\left(t\right)=E\left\{h_{q}\left(t\right)|W;\Delta^{\left(r,q-1\right)}\right\}\\ & = & b\left(\xi_{q}^{\left(r,q-1\right)}\right)m_{q}^{\left(r,q-1\right)}\left(t\right)+\left[w\left(t\right)-B\left(\xi^{\left(r,q-1\right)}\right)m^{\left(r,q-1\right)}\left(t\right)\right], \end{array}$$

(52) $$\begin{array}{ccc} & \; & D\left\{h_{q}\left(t\right)|W;\Delta^{\left(r,q-1\right)}\right\}=0_{K}. \end{array}$$

#### 5.1.2. M-Step

Update the estimates of $\xi_{q}$, $m_{q}$, and τ by solving

(53) $$\begin{array}{c} \min_{\xi_{q}\in \Omega ,m_{q},\tau >0}K\log\left(\tau \right)+\frac{1}{\tau L}\sum_{t=1}^{L}{∥h_{q}^{\left(r\right)}\left(t\right)-b\left(\xi_{q}\right)m_{q}\left(t\right)∥}^{2}. \end{array}$$

$\xi_{q}^{\left(r\right)}$, $m_{q}^{\left(r\right)}$, and $\tau^{\left(r,q\right)}$ are obtained by

(54) $$\begin{array}{ccc} \xi_{q}^{\left(r\right)} & = & \xi_{q}^{\left(r,q\right)}=\arg\max_{\xi_{q}\in \Omega }\mathrm{Tr}\left\{\Gamma_{q}{\hat{P}}_{q}^{\left(r\right)}\right\}, \end{array}$$

(55) $$\begin{array}{ccc} m_{q}^{\left(r\right)}\left(t\right) & = & m_{q}^{\left(r,q\right)}\left(t\right)=b^{H}\left(\xi_{q}^{\left(r\right)}\right)h_{q}^{\left(r\right)}\left(t\right)/K,\forall t, \end{array}$$

(56) $$\begin{array}{ccc} \tau^{\left(r,q\right)} & = & d_{q}^{\left(r\right)}/K, \end{array}$$

where ${\hat{P}}_{q}^{\left(r\right)}=\left(1/L\right)\sum_{t=1}^{L}h_{q}^{\left(r\right)}\left(t\right){\left[h_{q}^{\left(r\right)}\left(t\right)\right]}^{H}$ and $d_{q}^{\left(r\right)}=\mathrm{Tr}\left(\left(I_{K}-\Gamma_{q}^{\left(r\right)}\right){\hat{P}}_{q}^{\left(r\right)}\right)$.

Moreover, the other parameter estimates are not updated at this cycle and their iterative values are

(57) $$\begin{array}{ccc} \xi_{g}^{\left(r,q\right)} & = & \xi_{g}^{\left(r,q-1\right)},\forall g\neq q, \end{array}$$

(58) $$\begin{array}{ccc} m_{g}^{\left(r,q\right)}\left(t\right) & = & m_{g}^{\left(r,q-1\right)}\left(t\right),\forall g\neq q,t. \end{array}$$

**Remark** **2.** *Since the $h_{q}^{\left(r\right)}\left(t\right)$’s in (51), $\xi_{q}^{\left(r\right)}$ in (54), and the $m_{q}^{\left(r\right)}\left(t\right)$’s in (55) are unrelated to $\tau^{\left(r,q-1\right)}$, we can omit (56) due to the nuisance parameter τ.*

### 5.2. Random Signal Model

Based on (48), we have $h_{q}\left(t\right)∼\mathrm{CN}\left(0,N_{q}\right)$ with $N_{q}=\rho_{q}b\left(\xi_{q}\right)b^{H}\left(\xi_{q}\right)+\tau I_{K}$. However, the distribution of $h_{g}\left(t\right)$ with g≠q is only associated with $m_{g}\left(t\right)$. The complete-data LLF is written as

(59) $$\begin{array}{ccc} Z(\xi_{q},\rho ,\tau ) & = & \sum_{t=1}^{L}\sum_{g\neq q}\log p\left(m_{g}\left(t\right);\rho_{g}\right)+\sum_{t=1}^{L}\log p\left(h_{q}\left(t\right);\xi_{q},\rho_{q},\tau \right)\\ & = & -L\left(G-1\right)\log\left(\pi \right)-L\sum_{g\neq q}\left[\log\left(\rho_{g}\right)+{\hat{P}}_{g}/\rho_{g}\right]\\ & \; & -LK\log\left(\pi \right)-L\left[\log\left(\mathrm{Det}(N_{q})\right)+\mathrm{Tr}\left(N_{q}^{-1}{\hat{P}}_{q}\right)\right], \end{array}$$

where ${\hat{P}}_{g}=\left(1/L\right)\sum_{t=1}^{L}{\left|m_{g}\left(t\right)\right|}^{2}$ and |·| denotes modulus.

#### 5.2.1. E-Step

Calculate the conditional expectation of the complete-data LLF in (59)

(60) $$\begin{array}{ccc} E\left\{Z\left(\xi_{q},\rho ,\tau \right)|W;\Delta^{\left(r,q-1\right)}\right\} & = & V-L\sum_{g\neq q}\left[\log\left(\rho_{g}\right)+{\hat{P}}_{g}^{\left(r,q\right)}/\rho_{g}\right]\\ & \; & -L\left[\log\left(\mathrm{Det}(N_{q})\right)+\mathrm{Tr}\left(N_{q}^{-1}{\hat{P}}_{q}^{\left(r\right)}\right)\right], \end{array}$$

where Δ=(ξ,ρ,τ) and V=−L(K+G−1)log(π). In (60),

(61) $$\begin{array}{ccc} {\hat{P}}_{g}^{\left(r,q\right)} & = & E\left\{{\hat{P}}_{g}|W;\Delta^{\left(r,q-1\right)}\right\}\\ & = & \rho_{g}^{\left(r,q-1\right)}\left[1-b^{H}\left(\xi_{g}^{\left(r,q-1\right)}\right)d_{g}^{\left(r,q-1\right)}\right]+{\left[d_{g}^{\left(r,q-1\right)}\right]}^{H}{\hat{P}}_{w}d_{g}^{\left(r,q-1\right)}\geq 0 \end{array}$$

with $d_{g}^{\left(r,q-1\right)}={\left[N_{w}^{\left(r,q-1\right)}\right]}^{-1}b\left(\xi_{g}^{\left(r,q-1\right)}\right)\rho_{g}^{\left(r,q-1\right)}$ and

(62) $$\begin{array}{ccc} {\hat{P}}_{q}^{\left(r\right)} & = & {\hat{P}}_{q}^{\left(r,q\right)}=E\left\{{\hat{P}}_{q}|W;\Delta^{\left(r,q-1\right)}\right\}\\ & = & N_{q}^{\left(r,q-1\right)}-N_{q}^{\left(r,q-1\right)}{\left[N_{w}^{\left(r,q-1\right)}\right]}^{-1}N_{q}^{\left(r,q-1\right)}\\ & \; & +N_{q}^{\left(r,q-1\right)}{\left[N_{w}^{\left(r,q-1\right)}\right]}^{-1}{\hat{P}}_{w}{\left[N_{w}^{\left(r,q-1\right)}\right]}^{-1}N_{q}^{\left(r,q-1\right)}. \end{array}$$

#### 5.2.2. M-Step

Update the estimates of $\xi_{q}$, ρ, and τ by solving

(63) $$\begin{array}{c} \min_{\xi_{q}\in \Omega ,\rho \geq 0,\tau >0}\sum_{g\neq q}\left[\log\left(\rho_{g}\right)+{\hat{P}}_{g}^{\left(r,q\right)}/\rho_{g}\right]+\log\left(\mathrm{Det}(N_{q})\right)+\mathrm{Tr}\left(N_{q}^{-1}{\hat{P}}_{q}^{\left(r\right)}\right). \end{array}$$

We, thus, have $\rho_{g}^{\left(r,q\right)}={\hat{P}}_{g}^{\left(r,q\right)},\forall g\neq q$, and $\xi_{q}^{\left(r\right)}$, $\rho_{q}^{\left(r,q\right)}$, and $\tau^{\left(r,q\right)}$ are obtained by solving

(64) $$\begin{array}{c} \min_{\xi_{q}\in \Omega ,\sigma_{q}\geq 0,\tau >0}K\log\left(\tau \right)+\log\left(\mathrm{Det}(Q_{q})\right)+\frac{1}{\tau }\mathrm{Tr}\left(Q_{q}^{-1}{\hat{P}}_{q}^{\left(r\right)}\right), \end{array}$$

where $N_{q}=\tau Q_{q}$ with $Q_{q}=\sigma_{q}b\left(\xi_{q}\right)b^{H}\left(\xi_{q}\right)+I_{K}$ and $\sigma_{q}=\rho_{q}/\tau$. Following (45)–(47), $\xi_{q}^{\left(r\right)}$, $\rho_{q}^{\left(r,q\right)}$, and $\tau^{\left(r,q\right)}$ are obtained by

(65) $$\begin{array}{ccc} \xi_{q}^{\left(r\right)} & = & \xi_{q}^{\left(r,q\right)}=\arg\max_{\xi_{q}\in \Omega }\mathrm{Tr}\left(\Gamma_{q}{\hat{P}}_{q}^{\left(r\right)}\right), \end{array}$$

(66) $$\begin{array}{ccc} \tau^{\left(r,q\right)} & = & \left\{\begin{array}{cc} d_{q}^{\left(r\right)}/\left(K-1\right)\geq 0, & e_{q}^{\left(r\right)}>\mathrm{Tr}\left({\hat{P}}_{q}^{\left(r\right)}\right)/K,\\ \mathrm{Tr}\left({\hat{P}}_{q}^{\left(r\right)}\right)/K\geq 0, & e_{q}^{\left(r\right)}\leq \mathrm{Tr}\left({\hat{P}}_{q}^{\left(r\right)}\right)/K, \end{array}\right. \end{array}$$

(67) $$\begin{array}{ccc} \rho_{q}^{\left(r,q\right)} & = & \max\left\{\left(e_{q}^{\left(r\right)}-\mathrm{Tr}\left({\hat{P}}_{q}^{\left(r\right)}\right)/K\right)/\left(K-1\right),0\right\}, \end{array}$$

where $\tau^{\left(r,q\right)}=0$ is possible although its probability is very low. For example, if G=2, L=1, and $\rho_{1}^{\left(1,1\right)}=0$ at 1st cycle of the 1st iteration, at the 2nd cycle of the 1st iteration we will have

(68) $$\begin{array}{cc} & N_{w}^{\left(1,1\right)}=\rho_{2}^{\left(1,1\right)}b\left(\xi_{2}^{\left(1,1\right)}\right)b^{H}\left(\xi_{2}^{\left(1,1\right)}\right)+\tau^{\left(1,1\right)}I_{K}=N_{2}^{\left(1,1\right)} \end{array}$$

and then ${\hat{P}}_{2}^{\left(1\right)}=w\left(1\right)w^{H}\left(1\right)$ by (62). Furthermore, if $w\left(1\right)=hb\left(\bar{\xi }\right)$$\left(h\neq 0,\bar{\xi }\in \Omega \right)$, we will obtain $\xi_{2}^{\left(1\right)}=\bar{\xi }$ by (65) and $\tau^{\left(1,2\right)}=d_{2}^{\left(1\right)}/\left(K-1\right)=0$ by (66).

To avoid $\tau^{\left(r,q\right)}=0$, we use the following two CM-steps to re-estimate $\xi_{q}$, $\rho_{q}$, and τ based on problem (64) if $\tau^{\left(r,q\right)}=0$ in (66).

- *First CM-step:* Estimate $\xi_{q}$ and $\rho_{q}$ but hold $\tau =\tau^{\left(r,q-1\right)}$ fixed. Then, problem (64) is simplified to
  (69) $$\begin{array}{c} \min_{\xi_{q}\in \Omega ,\sigma_{q}\geq 0}\log\left(\mathrm{Det}(Q_{q})\right)+\frac{1}{\tau^{\left(r,q-1\right)}}\mathrm{Tr}\left(Q_{q}^{-1}{\hat{P}}_{q}^{\left(r\right)}\right). \end{array}$$
  Following (21) and (22), $\xi_{q}^{\left(r\right)}$ and $\rho_{q}^{\left(r,q\right)}$ are obtained by
  (70) $$\begin{array}{ccc} \xi_{q}^{\left(r\right)} & = & \xi_{q}^{\left(r,q\right)}=\arg\max_{\xi_{q}\in \Omega }\mathrm{Tr}\left(\Gamma_{q}{\hat{P}}_{q}^{\left(r\right)}\right), \end{array}$$
  (71) $$\begin{array}{ccc} \rho_{q}^{\left(r,q\right)} & = & \sigma_{q}^{\left(r,q\right)}\tau^{\left(r,q-1\right)}=\max\left\{\left(e_{q}^{\left(r\right)}-\tau^{\left(r,q-1\right)}\right)/K,0\right\}. \end{array}$$
- *Second CM-step:* Estimate τ but hold $\xi_{q}=\xi_{q}^{\left(r\right)}$ and $\sigma_{q}=\sigma_{q}^{\left(r,q\right)}$ fixed. Then, problem (64) is simplified to
  (72) $$\begin{array}{c} \min_{\tau >0}K\log\left(\tau \right)+\frac{1}{\tau }\mathrm{Tr}\left({\left[Q_{q}^{\left(r\right)}\right]}^{-1}{\hat{P}}_{q}^{\left(r\right)}\right), \end{array}$$
  where $Q_{q}^{\left(r\right)}=\sigma_{q}^{\left(r,q\right)}b\left(\xi_{q}^{\left(r\right)}\right)b^{H}\left(\xi_{q}^{\left(r\right)}\right)+I_{K}$. We obtain $\tau^{\left(r,q\right)}$ by
  (73) $$\begin{array}{cc} & \tau^{\left(r,q\right)}=\mathrm{Tr}\left\{{\left[Q_{q}^{\left(r\right)}\right]}^{-1}{\hat{P}}_{q}^{\left(r\right)}\right\}/K=\left(\tau^{\left(r,q-1\right)}+d_{q}^{\left(r\right)}\right)/K, \end{array}$$
  where $d_{q}^{\left(r\right)}=\mathrm{Tr}\left(\left(I_{K}-\Gamma_{q}^{\left(r\right)}\right){\hat{P}}_{q}^{\left(r\right)}\right)\geq 0$ and $\tau^{\left(r,q\right)}>0$ if $\tau^{\left(r,q-1\right)}>0$.

Moreover, the other parameter estimate(s) is (are) not updated at this cycle and the iterative value(s) is (are)

(74) $$\begin{array}{cc} & \xi_{g}^{\left(r,q\right)}=\xi_{g}^{\left(r,q-1\right)},\forall g\neq q. \end{array}$$

## 6. Properties of the Proposed EM, MEM, and SAGE Algorithms

### 6.1. Convergence Point

It is easy to verify that the above EM-type algorithms satisfy standard regularity conditions [5,21,26] and always converge to stationary points of J(Ψ,τ). Of course, the convergence points of these EM-type algorithms depend on their initial points. To generate appropriate initial points, we can employ the method presented in [10] using the deterministic signal model.

### 6.2. Complexity and Stability

At the *r*th iteration, the computational burdens of the above EM-type algorithms lie in solving the *G* maximization problems

(75) $$\begin{array}{c} \xi_{g}^{\left(r\right)}=\arg\max_{\xi_{g}\in \Omega }\mathrm{Tr}\left(\Gamma_{g}{\hat{P}}_{g}^{\left(r\right)}\right),\forall g. \end{array}$$

Thus, these EM-type algorithms have almost the same computational complexity at every iteration and if an algorithm has faster convergence, its number of iterations required will be smaller and its computations will be fewer.

However, when the powers of sources are unequal, we have found via simulation that the DOA estimates of multiple sources, updated by (75), tend to be consistent with the true DOA of the source with the largest power. Accordingly, these EM-type algorithms may be unstable. To address this issue, we can reduce the difference between $\xi_{g}^{\left(r\right)}$ and $\xi_{g}^{\left(r-1\right)}$ by increasing $\mathrm{Tr}\left(\Gamma_{g}{\hat{P}}_{g}^{\left(r\right)}\right)$ rather than maximizing it, i.e.,

(76) $$\begin{array}{c} \mathrm{Tr}\left(\Gamma_{g}^{\left(r\right)}{\hat{P}}_{g}^{\left(r\right)}\right)\geq \mathrm{Tr}\left(\Gamma_{g}^{\left(r-1\right)}{\hat{P}}_{g}^{\left(r\right)}\right),\forall g, \end{array}$$

which guarantees the monotonicity of these EM-type algorithms [3]. As a good example, Algorithm 1 in the next section has given excellent simulation results.
**Algorithm 1** Gradient ascent with backtracking line search1:$y\left(\eta_{g}\right)=K\times \mathrm{Tr}\left(\Gamma_{g}{\hat{P}}_{g}^{\left(r\right)}\right)$, $\eta_{g}=\eta_{g}^{\left(r-1\right)}\in \left(0,\pi \right)\;\left(\mathrm{radian}\right)$.2:**while** $|y^{\prime }\left(\eta_{g}\right)|>0.001$ **do**3:   $s=0.1\times \left\{\begin{array}{cc} \left(\pi -\eta_{g}\right)/y^{\prime }\left(\eta_{g}\right), & y^{\prime }\left(\eta_{g}\right)>0,\\ -\eta_{g}/y^{\prime }\left(\eta_{g}\right), & y^{\prime }\left(\eta_{g}\right)<0. \end{array}\right.$4:   **while** $y\left(\eta_{g}+sy^{\prime }\left(\eta_{g}\right)\right)<y\left(\eta_{g}\right)+0.3s{\left(y^{\prime }\left(\eta_{g}\right)\right)}^{2}$ **do**5:     s=0.5s.6:   **end while**7:   $\eta_{g}=\eta_{g}+sy^{\prime }\left(\eta_{g}\right)\in \left(0,\pi \right)\;\left(\mathrm{radian}\right)$.8:**end while**9:$\eta_{g}^{\left(r\right)}=\eta_{g}$.

## 7. Simulation Results

We give simulation results to compare the proposed EM-type algorithms. For convenience, the array is assumed to be a uniformly spaced linear array, in which $\zeta_{k}={\left[\frac{\chi }{2}\left(k-1\right)\;0\;0\right]}^{T}$. We set G=2, L=20, and β=1/G in the EM algorithm. Here, $\mu_{1}=\mu_{2}=90{}^{\circ}$ is known while $\eta_{1}$ and $\eta_{2}$ need to be estimated. M in the deterministic signal model is also generated by the independent random numbers $m_{g}\left(t\right)∼\mathrm{CN}\left(0,\rho_{g}\right)$. All the algorithms adopt the stopping criterion $∥\xi^{\left(r+1\right)}-\xi^{\left(r\right)}∥\leq 0.001{}^{\circ}$. Algorithm 1 is designed to search the $\eta_{g}^{\left(r\right)}$’s in (76) [27]. Moreover, $M^{\left(0\right)}={\left[1\;1\right]}^{T}$, $\rho^{\left(0\right)}=1$, $\tau^{\left(0\right)}=1/G$, and $\tau^{\left(0\right)}=1$. In this section, the EM, MEM, and SAGE algorithms are simply written as EM, MEM, and SAGE, respectively.

### 7.1. Deterministic Signal Model

**Figure 1:**
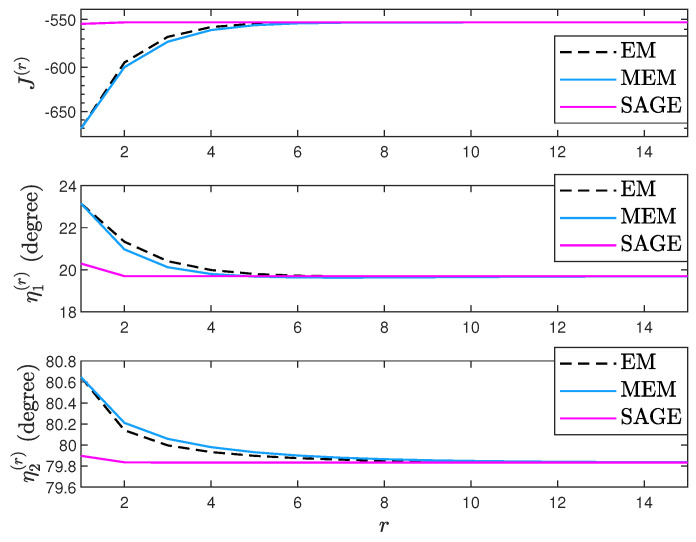
$J(\Psi^{\left(r\right)},\tau^{\left(r\right)})$, $\eta_{1}^{\left(r\right)}$, and $\eta_{2}^{\left(r\right)}$ comparison.

To compare the convergence of EM, MEM, and SAGE, Figure 1 plots their $J(\Psi^{\left(r\right)},\tau^{\left(r\right)})$’s, $\eta_{1}^{\left(r\right)}$’s, and $\eta_{2}^{\left(r\right)}$’s under one trial. In Figure 1, K=10, $\eta_{1}=20{}^{\circ}$, $\eta_{2}=80{}^{\circ}$, $\rho_{1}=-2\;\mathrm{dB}$, $\rho_{2}=4\;\mathrm{dB}$, τ=4dB, $\eta_{1}^{\left(0\right)}=24{}^{\circ}$, and $\eta_{2}^{\left(0\right)}=84{}^{\circ}$. It is easy to see that EM, MEM, and SAGE converge to a consistent $\left(\eta_{1},\eta_{2}\right)$ estimate given an accurate initial point. Moreover, EM has a similar convergence with MEM while SAGE converges faster than EM and MEM.

**Figure 2:**
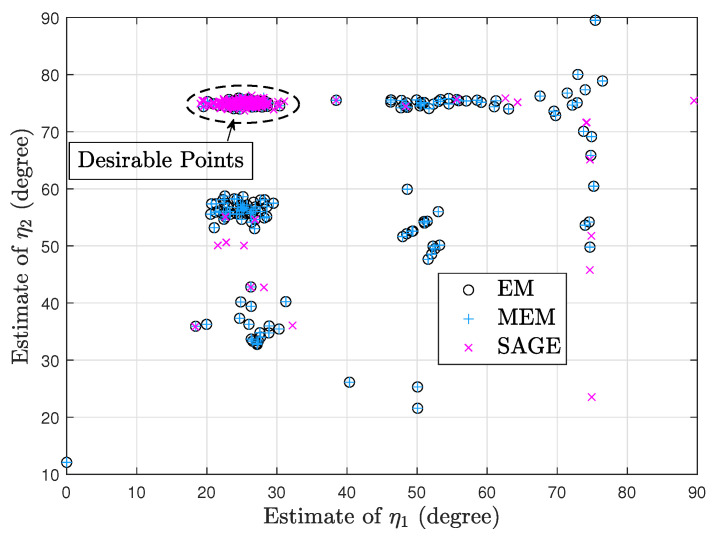
Scatter plot of $\left(\eta_{1},\eta_{2}\right)$ estimates.

**Figure 3:**
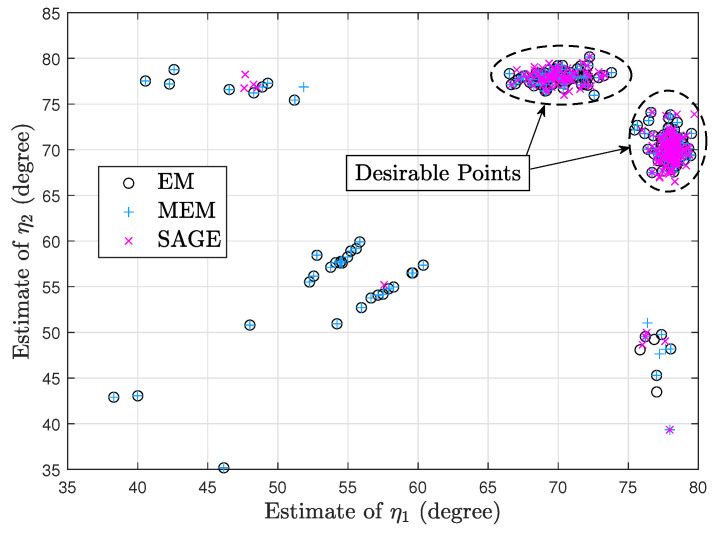
Scatter plot of $\left(\eta_{1},\eta_{2}\right)$ estimates.

Figure 2 and Figure 3 show two scatter plots of $\left(\eta_{1},\eta_{2}\right)$ estimates under 200 trials. In Figure 2, K=10, $\eta_{1}=25{}^{\circ}$, $\eta_{2}=75{}^{\circ}$, $\rho_{1}=-4\;\mathrm{dB}$, $\rho_{2}=2\;\mathrm{dB}$, τ=4dB, $\eta_{1}^{\left(0\right)}=40{}^{\circ}$, and $\eta_{2}^{\left(0\right)}=60{}^{\circ}$. Moreover, the numbers of desirable points obtained by EM, MEM, and SAGE are 68, 72, and 179, respectively. In Figure 3, K=10, $\eta_{1}=70{}^{\circ}$, $\eta_{2}=78{}^{\circ}$, $\rho_{1}=-2\;\mathrm{dB}$, $\rho_{2}=4\;\mathrm{dB}$, τ=4dB, $\eta_{1}^{\left(0\right)}=50{}^{\circ}$, and $\eta_{2}^{\left(0\right)}=58{}^{\circ}$. Moreover, the numbers of desirable points obtained by EM, MEM, and SAGE are 159, 157, and 190, respectively. Figure 2 and Figure 3 imply that given a poor initial point, SAGE can avoid the convergence to an undesirable stationary point of J(Ψ,τ) more efficiently than EM and MEM.

Note that in each of Figure 2 and Figure 3, the SAGE algorithm requires the least processing time due to the fastest convergence and thus performs the fewest computations required. Moreover, both sources in Figure 2 are not closely spaced, so it is very difficult to mix up both sources and the desirable points in Figure 2 are centered around the true position (25°,75°). However, both sources in Figure 3 are closely spaced and the desirable points are centered around (78°,70°) or (70°,78°), i.e., these EM-type algorithms are likely to mix up closely spaced sources.

According to these simulations, we can conclude that (1) EM has similar convergence with MEM, and (2) SAGE outperforms EM and MEM.

### 7.2. Random Signal Model

**Figure 4:**
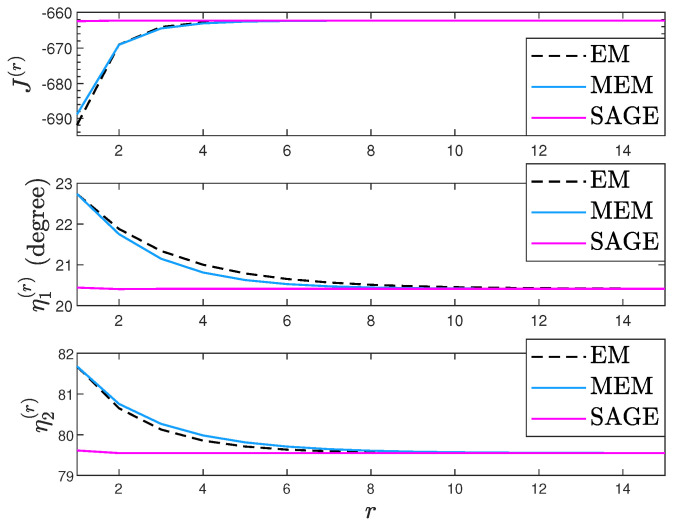
$J(\Psi^{\left(r\right)},\tau^{\left(r\right)})$, $\eta_{1}^{\left(r\right)}$, and $\eta_{2}^{\left(r\right)}$ comparison.

To compare the convergence of EM, MEM, and SAGE, Figure 4 plots their $J(\Psi^{\left(r\right)},\tau^{\left(r\right)})$’s, $\eta_{1}^{\left(r\right)}$’s, and $\eta_{2}^{\left(r\right)}$’s under one trial. In Figure 4, K=10, $\eta_{1}=20{}^{\circ}$, $\eta_{2}=80{}^{\circ}$, $\rho_{1}=-4\;\mathrm{dB}$, $\rho_{2}=4\;\mathrm{dB}$, τ=4dB, $\eta_{1}^{\left(0\right)}=24{}^{\circ}$, and $\eta_{2}^{\left(0\right)}=84{}^{\circ}$. We can also observe that given an accurate initial point, EM, MEM, and SAGE converge to a consistent $\left(\eta_{1},\eta_{2}\right)$ estimate. Moreover, EM has similar convergence with MEM while SAGE converges faster than EM and MEM.

**Figure 5:**
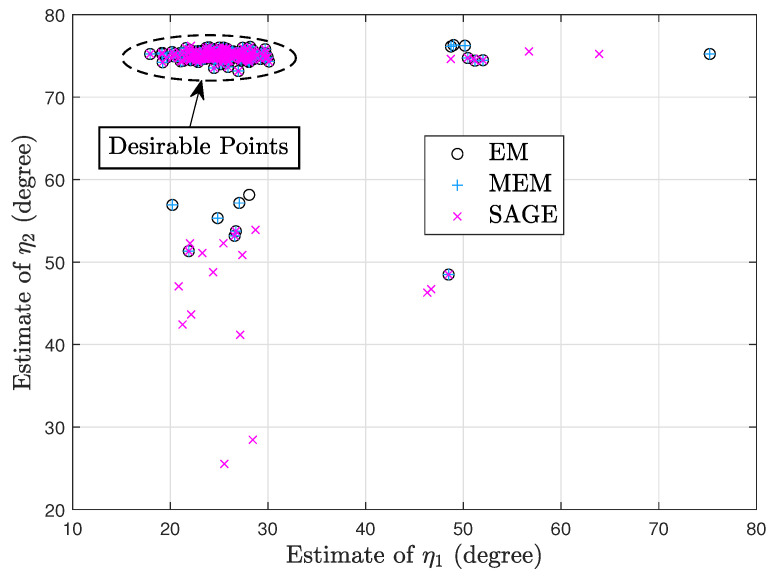
Scatter plot of $\left(\eta_{1},\eta_{2}\right)$ estimates.

**Figure 6:**
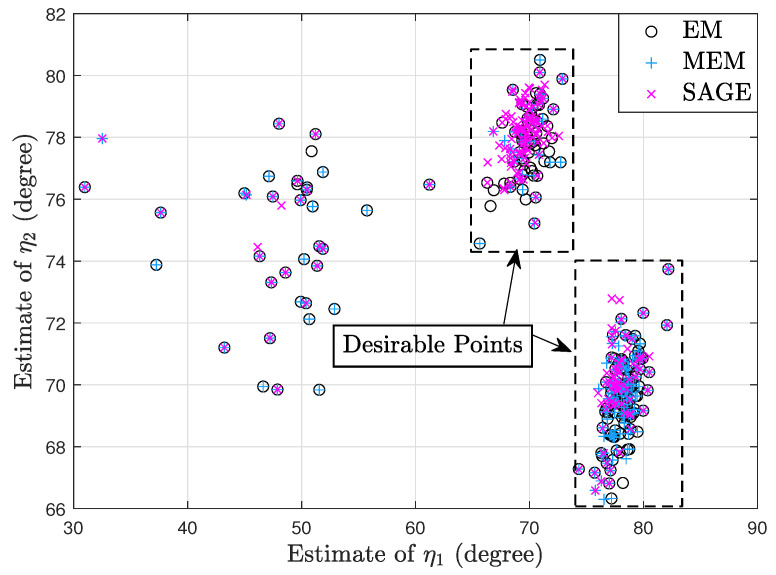
Scatter plot of $\left(\eta_{1},\eta_{2}\right)$ estimates.

Figure 5 and Figure 6 show two scatter plots of $\left(\eta_{1},\eta_{2}\right)$ estimates under 200 trials. In Figure 5, K=10, $\eta_{1}=25{}^{\circ}$, $\eta_{2}=75{}^{\circ}$, $\rho_{1}=-4\;\mathrm{dB}$, $\rho_{2}=2\;\mathrm{dB}$, τ=4dB, $\eta_{1}^{\left(0\right)}=40{}^{\circ}$, and $\eta_{2}^{\left(0\right)}=60{}^{\circ}$. Moreover, the numbers of desirable points obtained by EM, MEM, and SAGE are 185, 186, and 175, respectively. In Figure 6, K=10, $\eta_{1}=70{}^{\circ}$, $\eta_{2}=78{}^{\circ}$, $\rho_{1}=-2\;\mathrm{dB}$, $\rho_{2}=-1\;\mathrm{dB}$, τ=4dB, $\eta_{1}^{\left(0\right)}=55{}^{\circ}$, and $\eta_{2}^{\left(0\right)}=63{}^{\circ}$. Moreover, the numbers of desirable points obtained by EM, MEM, and SAGE are 161, 161, and 172, respectively. Figure 5 and Figure 6 imply that EM has similar convergence with MEM but compared to EM and MEM, SAGE is less and more efficient for avoiding the convergence to an undesirable stationary point of J(Ψ,τ) in Figure 5 and Figure 6, respectively. Note that in each of Figure 5 and Figure 6, the SAGE algorithm requires the least processing time due to the fastest convergence and thus performs the fewest computations required. In addition, these EM-type algorithms are likely to mix up closely spaced sources, so the desirable points in Figure 5 are centered around (25°,75°) and the desirable points in Figure 6 are centered around (78°,70°) or (70°,78°).

Based on the above figures, we can conclude that (1) EM has similar convergence with MEM, and (2) SAGE cannot always outperform EM and MEM.

### 7.3. Deterministic and Random Signal Models

Snapshots from the random signal model can be processed by these EM-type algorithms for the deterministic signal model, which means that we can compare these algorithms for both signal models. The above simulation results have shown that EM has similar convergence with MEM, so we only compare EM and SAGE for both signal models in this subsection for simplicity.

**Figure 7:**
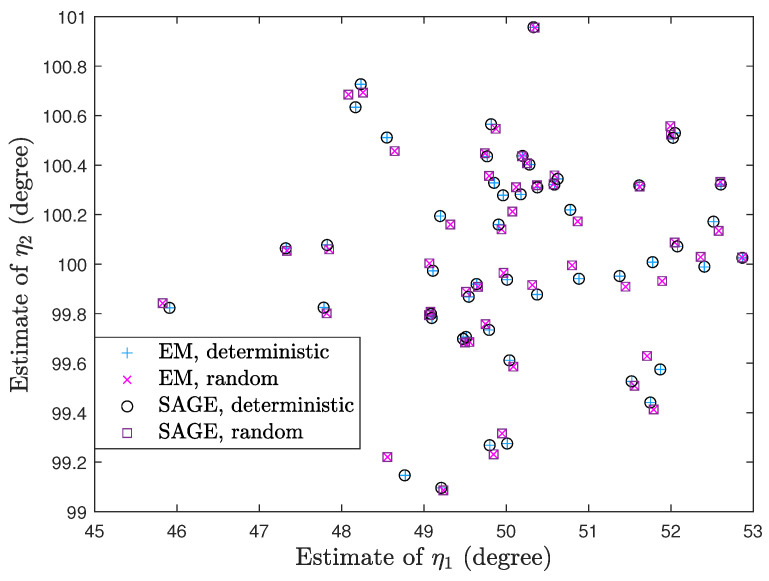
Scatter plot of $\left(\eta_{1},\eta_{2}\right)$ estimates.

Since both signal models have the same DOA parameter ξ, the stopping criterion $∥\xi^{\left(r+1\right)}-\xi^{\left(r\right)}∥\leq 0.001{}^{\circ}$ is suitable. Figure 7 shows a scatter plot of $\left(\eta_{1},\eta_{2}\right)$ estimates under 50 trials. In Figure 7, K=10, $\eta_{1}=50{}^{\circ}$, $\eta_{2}=100{}^{\circ}$, $\rho_{1}=-4\;\mathrm{dB}$, $\rho_{2}=4\;\mathrm{dB}$, τ=4dB, $\eta_{1}^{\left(0\right)}=55{}^{\circ}$, and $\eta_{2}^{\left(0\right)}=95{}^{\circ}$. We can see that EM and SAGE for both signal models yield close $\left(\eta_{1},\eta_{2}\right)$ estimates for each trial.

**Figure 8:**
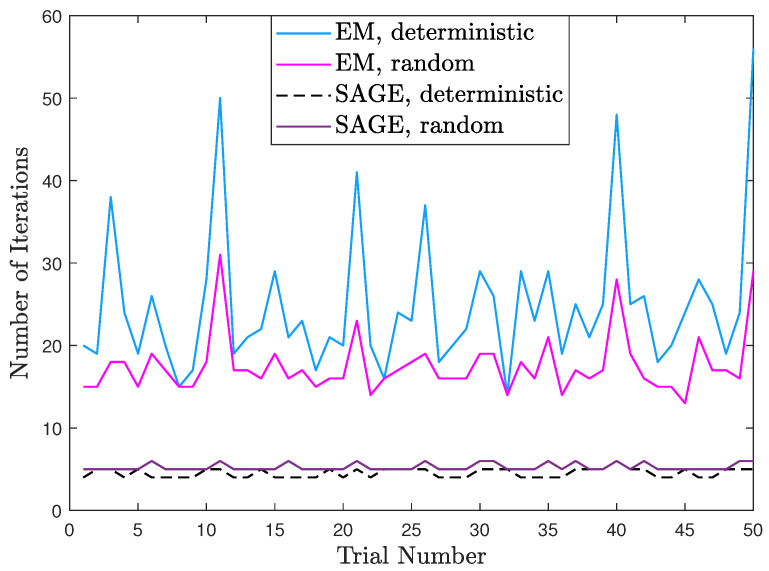
Numbers of iterations of different algorithms.

Based on Figure 7, Figure 8 compares the numbers of iterations required by these algorithms. We can observe that EM for the deterministic signal model generally needs more iterations than EM for the random signal model. The reason is that EM for deterministic signal model needs to update more parameter estimates at each iteration and, thus, has slower convergence than EM for the random signal model. Moreover, SAGE for the deterministic signal model generally needs fewer iterations than SAGE for the random signal model. More importantly, SAGE for the deterministic signal model always requires the fewest iterations for each trial. Thus, we can conclude that SAGE for the deterministic signal model is superior to the other algorithms in the computational cost.

**Figure 9:**
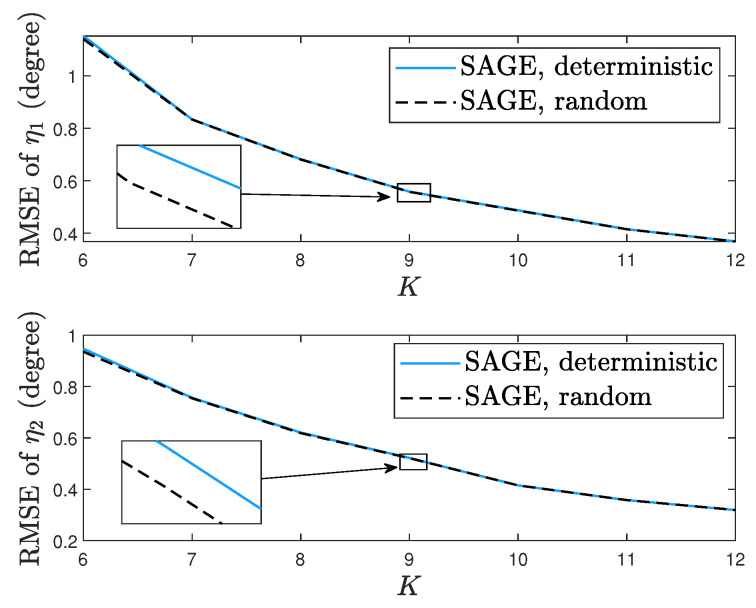
RMSEs of the SAGE algorithm.

Figure 9 shows the root mean square error (RMSE) performances of DOA estimation obtained by the SAGE algorithm for the deterministic and random signal models. In Figure 9, $\eta_{1}=60{}^{\circ}$, $\eta_{2}=120{}^{\circ}$, $\rho_{1}=0\;\mathrm{dB}$, $\rho_{2}=1\;\mathrm{dB}$, τ=3dB, $\eta_{1}^{\left(0\right)}=55{}^{\circ}$, and $\eta_{2}^{\left(0\right)}=115{}^{\circ}$. Each RMSE is computed from 1000 independent realizations. We can observe that as the number of sensors *K* increases, the SAGE algorithm for each signal model yields small RMSEs, which indicates that increasing the number of sensors *K* can improve the accuracy of DOA estimation.

## 8. Conclusions

In this paper, we applied and designed the EM and SAGE algorithms for DOA estimation in unknown uniform noise and proposed a new MEM algorithm applicable to the noise assumption. Next, we improved these EM-type algorithms to ensure the stability when the powers of sources are unequal. After being improved, simulation results illustrated that the EM algorithm has similar convergence with the MEM algorithm, the SAGE algorithm outperforms the EM and MEM algorithms for the deterministic signal model, and the SAGE algorithm cannot always outperform the EM and MEM algorithms for the random signal model. In addition, simulation results indicated that when these EM-type algorithms process the same snapshots from the random signal model, the SAGE algorithm for the deterministic signal model can require the fewest iterations and computations.

## Data Availability

The data presented in this study are available on request from the corresponding author. The data are not publicly available, due to the data in this paper not being from publicly available datasets but obtained from the simulation of the signal models listed in the paper.
